# Discovery of novel human transcript variants by analysis of intronic single-block EST with polyadenylation site

**DOI:** 10.1186/1471-2164-10-518

**Published:** 2009-11-12

**Authors:** Pingzhang Wang, Peng Yu, Peng Gao, Taiping Shi, Dalong Ma

**Affiliations:** 1 Chinese National Human Genome Center, #3-707 North YongChang Road BDA, Beijing 100176, PR China; 2 Laboratory of Medical Immunology, School of Basic Medical Science, Peking University Health Science Center, 38# Xueyuan Road, Beijing, 100083, PR China; 3 Peking University Center for Human Disease Genomics, 38# Xueyuan Road, Beijing, 100083, PR China

## Abstract

**Background:**

Alternative polyadenylation sites within a gene can lead to alternative transcript variants. Although bioinformatic analysis has been conducted to detect polyadenylation sites using nucleic acid sequences (EST/mRNA) in the public databases, one special type, single-block EST is much less emphasized. This bias leaves a large space to discover novel transcript variants.

**Results:**

In the present study, we identified novel transcript variants in the human genome by detecting intronic polyadenylation sites. Poly(A/T)-tailed ESTs were obtained from single-block ESTs and clustered into 10,844 groups standing for 5,670 genes. Most sites were not found in other alternative splicing databases. To verify that these sites are from expressed transcripts, we analyzed the supporting EST number of each site, blasted representative ESTs against known mRNA sequences, traced terminal sequences from cDNA clones, and compared with the data of Affymetrix tiling array. These analyses confirmed about 84% (9,118/10,844) of the novel alternative transcripts, especially, 33% (3,575/10,844) of the transcripts from 2,704 genes were taken as high-reliability. Additionally, RT-PCR confirmed 38% (10/26) of predicted novel transcript variants.

**Conclusion:**

Our results provide evidence for novel transcript variants with intronic poly(A) sites. The expression of these novel variants was confirmed with computational and experimental tools. Our data provide a genome-wide resource for identification of novel human transcript variants with intronic polyadenylation sites, and offer a new view into the mystery of the human transcriptome.

## Background

Eukaryotic mRNA is frequently alternative spliced. Recent studies of human tissue transcriptomes by high-throughput sequencing have revealed that about 95% of multi-exon genes undergo alternative splicing (AS) [1,2]. This greatly enhances previous estimate of human AS events [3-5], thus further adds complexity to transcripts and proteins. Alternative cleavage and polyadenylation (APA) is also an important mechanism to produce diverse mRNA isoforms. In APA events, a key regulatory step in the formation of the mRNA 3'-end, a nascent mRNA is cleaved at its cleavage site and the poly(A) tail is added to the mRNA [6,7]. Polyadenylation is associated with important *cis*-elements, such as the upstream canonical AAUAAA and its hexamer variants, the downstream U/GU-rich elements, the auxiliary upstream elements, and the downstream elements [8-11]. These element combinations determine how mRNA 3'-ends are processed. In human, over half of genes have alternative polyadenylation products [9]. These alternative transcripts are often expressed in a tissue-specific pattern, and contribute to some inherited disorders and tumor development [12-16].

In addition to the 3' most exons, polyadenylation sites (poly(A) sites) can also exist in introns and internal exons. In human, at least 20% of the genes have intronic polyadenylation [17]. Alternative tandem or intronic poly(A) sites can lead to alternative polyadenylation [18]. Bioinformatic analysis has revealed different polyadenylation configuration within gene structure [11,17,19]. The mRNA produced from an internal polyadenylation site often encodes truncated proteins or distinct protein isoforms. These protein products often show different cellular localization and/or different functions compared to the protein produced from the 3'-most poly(A) site [20-29].

Genome-wide searches for poly(A) sites resulted in the polyA_DB and PolyA_DB2 (the latest version) databases [17,30,31]. To date, 54,686 poly(A) sites have been identified [31]. However, these poly(A) sites are mainly limited to coding regions, and the frequency of poly(A) sites in large introns and intergenic regions remains largely unstudied. In addition, the sequence selection for these databases was biased towards sequences in the UniGene database [30,32]. Because intron did not overlap with known exons or cDNA sequences, most intronic expressed sequence tag (EST) sequences were excluded. For example, ESTs located in large introns were removed in Lee's research [32], because these sequences usually did not overlap with other sequences for the same gene. No doubt, this bias leaves a large pool of undiscovered transcript variants with intronic polyadenylation sites.

To identify these underrepresented poly(A) sites, we preferentially selected intronic single-block ESTs considering that ESTs that span multiple exons often have been included in known UniGene clusters and have been used for study of alternative splicing. However, single-block ESTs, which span just one exon on chromosome, were not well considered [33-37]. We focused on the intronic 3'-end exon sites associated with poly(A/T)-tailed ESTs derived from single-block ESTs. An intronic 3'-end exon site is defined as a terminal exon site located in introns upstream of the 3'-most exon of the gene. Herein we use the term "3'-end exon site", but not "3'-end exon" to describe intronic poly(A/T)-tailed single-block ESTs because these 3'-end exons are usually incomplete at their 5'-ends and the closest exon junction is ambiguous. As a result, 10,844 intronic 3'-end exon sites from 5,670 human genes were identified. 45% of all these sites represent novel transcript variants that are absent from other alternative splicing related databases. To confirm that these sites are transcribed, we collected expression data from non-poly(A/T)-tailed ESTs, full-length cDNAs, end-pair sequencing of cDNA clones, and Affymetrix genomic tiling arrays. These data confirm that about 84% of the predicted sites represent true transcripts. We also successfully verified some predicted transcripts by RT-PCR experiments.

## Results

### Mapping and clustering intronic poly(A) sites in the human genome

**Figure 1:**
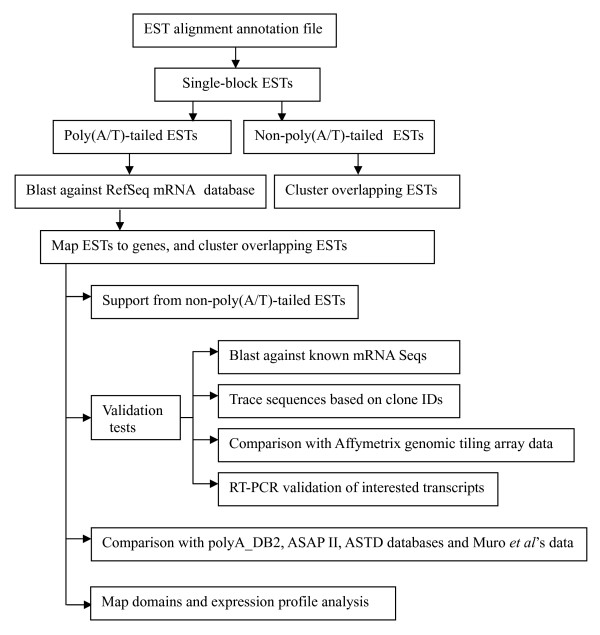
**A pipeline for identifying novel intronic 3'-end exon sites**. See Results, and Methods for details.

To identify novel transcript variants resulting from previously unidentified intronic poly(A) sites, an annotated EST alignment file from UCSC Genome Browser http://genome.ucsc.edu was analyzed (Figure 1). We focused on single-block ESTs that did not overlap known mRNA sequences. Initially, 7,948,198 aligned EST entries were analyzed for poly(A) sites. Among these, 3,614,581 single-block EST entries were obtained, containing 3,323,676 non-redundant ESTs. These ESTs could be further divided into two type: poly(A/T)-tailed or non-poly(A/T)-tailed, with the number of 494,529 and 2,829,147, respectively (Table 1). For the poly(A/T)-tailed ESTs, by blasting these sequences against the RefSeq mRNA database, known poly(A) sites were identified and removed, leaving 22,117 sequences for further analysis. These ESTs were finally clustered into 10,844 groups (poly(A) clusters) from 5,670 human genes (Table 1 and Additional file 1, sheet "all_site") according to their position overlapping in the chromosome alignment. These clusters represented 3'-end exon sites. Thus, the involved gene increased previous reported number that at least 3,344 human genes contained intronic poly(A) sites [17]. The various poly(A/T)-tailed ESTs in the same cluster may represent heterogeneous cleavage or different polyadenylation pattern if they contain different poly(A) sites [6,11]. Most 3'-end exon sites were flanked by exons containing coding sequences (CDS).

**Table 1:** Summary of novel human intronic 3'-end exon sites

	Site numbers	Genes	% all 3'-end exon sites
**i. Single-block ESTs**			
No. of poly(A/T)-tailed ESTs	494,529		
No. of non-poly(A/T)-tailed ESTs	2,829,147		
No. of final 3'-end exon sites	10,844	5,670	100%
No. supported by non-poly(A/T)-tailed ESTs	7,676	4,599	71%
**ii. Validation tests**			
Blast hit against all known cDNAs	2,957	2,257	27%
Support from EST clone IDs	1,155	1,001	11%
Support from Affymetrix transcripts	5,475	3,627	50%
RT-PCR experiments	10/26		
**iii. Overlapping with other AS and APA databases**			
PolyA_DB2	1,410	1,235	13%
ASAP II database	613	554	6%
ASTD database	1,250	1,115	11%
Muro *et al*'s data	4,046	2,895	37%
**iv. PAS analysis**			
PAS positive	7,051	4,292	65%
**v. Map domains**			
At least one domain deletion	7,641	4,142	70%
Transmembrane helix deletion	1,616	945	15%

See Results for details.

Among the single-block ESTs, 2,829,147 ESTs without poly(A/T)-tail were grouped in 396,094 clusters. Non-poly(A) clusters that overlapped with the poly(A/T)-tailed 3'-end exon clusters would support the expression of the novel transcripts. Of the intronic 3'-end exon sites, 7,676 (71%) from 4,599 genes had at least one supporting non-poly(A/T)-tailed EST (Additional file 2), and 3,041 (28%) of the poly(A) clusters contained at least two poly(A/T)-tailed ESTs (Additional file 1). Totally, 75% (8,189/10,844) of the identified 3'-end sites were supported by at least two ESTs. Among the resting 25% supported by only a single EST, 37% (974/2,655) got further supported by transcriptional data from Affymetrix genomic tiling array (see below, Additional file 1 and 2).

There were 351 independent poly(A) clusters that could overlap with their adjacent clusters via the bridge of non-poly(A) clusters. Some poly(A) clusters bridged several non-poly(A) clusters into a single large cluster (data not shown). These large clusters could just manifest the heterogeneity of the polyadenylation pattern at the 3'-end exons in the local genomic context [11,38].

### 3'-end novel transcript variants are expressed

To confirm that these poly(A) sites represent novel alternative transcript variants and not genomic DNA contamination, our analysis pipeline had four steps. First, we did blast searches against all known mRNAs excluding sequences from RefSeq. Second, for ESTs with clone ID, we traced their partner sequences of the same clones and checked for splicing signals within the sequences. Third, we compared the 3'-end exon sites with the data of Affymetrix tiling array. Finally, we selected some novel transcript variants and verified them via RT-PCR experiments.

In our analysis, poly(A/T)-tailed ESTs that had hits in the RefSeq mRNA database by blast searching were eliminated as known transcripts. However, there are many mRNAs are not included in the RefSeq database. Most of the sequences are produced by full-length cDNA sequencing projects. If our 3'-end ESTs could be aligned well to such cDNAs, the ESTs were thought to be potential novel transcripts. Among the 10,844 3'-end exon sites, 2,957 (27%, 2,957/10,844) from 2,257 genes had hits from at least one mRNA (Table 1 and Additional file 1). This indicates the transcript variants have been cloned by others. As the full-length cDNA sequencing projects have been conducted with state-of-art quality control as well as manual verification, it is appropriate that most of these supported ESTs stand for bona fide mRNAs. The remaining 7,887 sites, involving 3,413 human genes, may represent unidentified 3'-end exon sites for novel transcript variants.

ESTs often have clone IDs, which identify the plasmid clones of source cDNA fragments. EST sequences are produced from single-pass sequencing of 5'- and/or 3'-end of the clones. As we have got the 3'-end single-block ESTs, we could trace their corresponding 5'-end ESTs with the same clone IDs. If the 5'-end EST could be split into multiple blocks, with adjacent GT/AG splicing signal on the human genome, which could be taken as the exons in mature mRNAs, it was concluded that the pair of 5'-end and 3'-end ESTs comprised a bona fide mRNA.

In our data, 3'-end exon sites contained two types of ESTs: poly(A/T)-tailed and non-poly(A/T)-tailed but overlapped with the former. If either type of the ESTs had multi-block 5'-end ESTs, the 3'-end exon site was thought to be supported. First, traced sequences were obtained for the poly(A) ESTs, and 25 novel transcript variants were obtained (Additional file 3). None of the intronic poly(A/T)-tailed ESTs for the 25 clones were in PolyA_DB2 database. Second, traced sequences were also obtained for the non-poly(A) ESTs. From 185,278 non-poly(A/T)-tailed sequences, 134,658 clone IDs were extracted and 43,595 of these clones had multiple traced sequences. Of these traces sequences, 8,689 had multiple blocks aligned with the human genome with at least one block overlapped with RefSeq mRNAs. These sequences offered supporting evidence for novel 3'-end exons. Overall, EST clones provided evidence that 1,155 poly(A) sites from 1,001 genes represented expressed transcript variants (Table 1 and Additional file 2).

Transcriptional fragments from the Affymetrix genomic tiling array [39], which could support the existence of transcripts through the specified chromosome region, were integrated in our analysis. Affymetrix fragments overlapped with 5,475 3'-end exon sites (50%) from 3,627 genes (Table 1 and Additional file 2).

**Figure 2:**
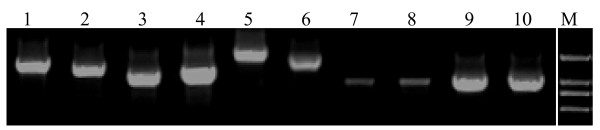
**RT-PCR confirms expression of predicted alternative transcript variants**. The novel transcript variants for 10 genes are illustrated. These genes are *DLL1 *(lane 1), *MAPK9 *(lane 2), *TNFRSF1A *(lane 3), and *STAMBP *(lane 4), *MAPK14 *(lane 5), *IL16 *(lane 6), *DGCR2 *(lane 7), *PDCD6IP *(lane 8), *PIAS1 *(lane 9) and *IL19 *(lane 10). Detail gene information is shown in Additional file 4. On the right are size markers (bp), which designate 2000 bp, 1500 bp, 750 bp, and 500 bp from the top down, respectively.

Finally, we selected novel isoforms of a couple of genes which have roles in signal transduction and did nested-PCR verification. Our interest was to explore the function of novel protein products encoded by the transcripts. It was expected that the full coding sequence should be included in PCR products. The primer strategy was that the upstream primer (5') was located nearby the translational start site (ATG) of the RefSeq mRNA, while the downstream primer (3') should be located in the poly(A/T)-tailed ESTs. Primer sets were listed in Additional file 4. The electrophoresis bands of the second PCR products were shown in Figure 2. Sequences of the PCR products were made blast search, and were revealed to be novel (Additional file 4). Sequence analysis was also made with the BLAT program.

**Figure 3:**
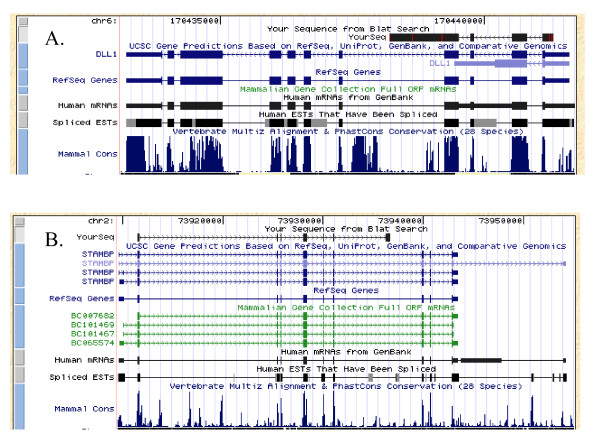
**Chromosomal alignment results for novel transcripts of *DLL1 *and *STAMBP***. The alignments for (A) *DLL1*, (B) *STAMBP*. Blocks show the exons, while the connecting lines depict the introns. Arrows in the connecting lines indicate the direction of transcription. The submitted sequences are indicated as "YourSeq" in each panel (A and B). A prolonged (A) or an additional block (B) relative to the reference sequences. These blocks represent prolonged or novel exons previously unidentified, that is, the "composite exons" and "hidden exons" [19].

As a result, RT-PCRs confirmed transcription of at least 38% (10 of 26 candidates) of selected intronic poly(A) sites. Acquired novel sequences and their accession numbers in GenBank database were listed in Additional file 4. In case of MAPK14 (Mitogen-activated protein kinase 14, also known as p38 alpha.), two novel alternative splicing variants were obtained, FJ032367 and FJ032368. The latter had an extra 27 nt resulting from alternative receptor site in exon 7, just like caspase-9 gamma [40], and a in-frame pre-stop codon is therefore introduced. The 3'-end exons that defined novel transcript variants could either be "hidden exons", not overlapping with any known exons, or "composite exons", extending known exons [19]. One "composite exon" and one "hidden exon" examples were shown in Figure 3A and 3B, respectively. The 3'-end exon for *DLL1 *(Delta-like 1) was "composite exon" (Figure 3A), whereas the pattern for *STAMBP *(STAM binding protein) was "hidden exon" (Figure 3B). The submitted sequences were indicated as "YourSeq" in each panel (Figure 3A and 3B). A prolonged (Figure 3A) or an additional block (Figure 3B) relative to the reference sequences was shown. These blocks represented prolonged or novel exons previously unidentified, that is, the "composite exons" and "hidden exons".

Further analysis of these transcript variants suggested that these variants complied with GT/AG rules and were not incompletely processed mRNAs. For the resting 16 candidates that were not successfully cloned, it might be relevant to limited tissue cDNA sources, unsuitable primers, PCR condition, low expression level of target transcripts, and non-specific amplification, and so on.

In summary, the majority (84%) of our poly(A) sites were supported by at least one of validation steps, as well as non-poly(A/T)-tailed ESTs (Additional file 2). Among these validation tests, the new isoforms supported by the RT-PCR, EST clone ID and blast hit against full cDNAs are more trustable than those only from Affymetrix validation because the single-block ESTs in the 3'-end exon sites can be joined to the upstream part of the annotated genes, whereas sites supported by Affymetrix tiling array may belong to independent genes hidden in the introns. Totally, 3,575 (33%, 3,575/10,844) 3'-end exon sites from 2,704 genes were taken as high-reliability (Additional file 1, sheet "validation_test"). The resting sites, whether they were supported by Affymetrix transcriptional fragments or not, were relatively less reliable and more dependent of experimental validation to exclude independently expressed transcripts. Among our 10 validated novel isoforms from RT-PCR experiments, 6 were still successfully cloned although their 3'-end exons were not supported by above mentioned EST clone ID and blast hit against full cDNAs.

The average length of the 3'-end exon sites in our result was 437 nucleotides, whereas the average length of the 3'-end exons of all the human RefSeqs was about 820 nucleotides. This could be explained by that ESTs just represent segments of complete transcriptional sequences, and therefore, a 3'-end exon site just represents partial sequence of a full 3'-end exon. No doubt, the actual 3'-end exon could extend toward upstream (5' end), and the precise nearest exon-exon boundary could be revealed. Theoretically, a 3'-end exon site corresponds to one full-length transcript which needs PCR validation to reveal the complete 3'-end exon. This is the reason that we would like to use the term "3'-end exon site", but not "3'-end exon" to describe these incomplete 3'-end exons in our study.

### Comparison with other alternative splicing related databases

To make a comparison with the PolyA_DB2 database, the accession numbers of poly(A/T)-tailed ESTs as well as the positions of chromosome alignment of each cluster were used. As a result, total 1,410 (13%) 3'-end exon sites from 1,235 genes were covered by PolyA_DB2 database (Table 1 and Additional file 1). Among some of these overlapping poly(A) sites, we found more supporting ESTs. For example, the poly(A) site in PolyA_DB2, Hs.279594.1.27, included only one poly(A/T)-tailed sequence (BQ772378), but in our dataset, the corresponding 3'-end exon site (ExonSiteNo is 3479) was supported by two poly(A/T)-tailed ESTs (BQ772378 and AW293188), and seven non-poly(A/T)-tailed ESTs (BF902676, BQ933237, DB119003, CR744722, AW805980, AI612802, and AW198031). It suggests from above analysis that our data can well complement previous studies.

Up to date, many alternative splicing databases have been developed [33-37,41,42], the main purpose is to collect all the alternative splicing candidates. It seems that one important common aspect for these databases is that multiple-block exons are used for analysis, and precise exon-intron boundary is required, whereas single-block ESTs are not well considered. We made a comparison between our data and two reputed alternative splicing databases, ASAP II database (released in 2007) [43] and ASTD (released in 2008) [44], which superseded the ASD (Alternative Splicing Database) [41] and ATD (Alternative Transcript Diversity) [45] databases. As shown in Table 1 and Additional file 1, among 10,844 3'-end exon sites, only 6% (613/10,844) sites from 554 genes were covered by ASAP II, whereas 11% (1,250/10,844) sites from 1,115 genes were covered by ASTD database (Table 1 and Additional file 1). This suggests that most of our data are novel.

During our process, Muro *et al *recently identified 3'-ends of human and murine genes by automated EST cluster analysis [46], we compared their data and ours, and found that about 37% (4,046/10,844) sites from 2,895 genes were same (Table 1 and Additional file 1). Excluding all the above crossing 3'-end exon sites and the sites having blast hit against full cDNAs, a total 45% (4,905/10,844) from 3,269 genes are novel and unique in our data.

### Novel transcript variants are derived from processed mature mRNAs

From the sequence analysis shown in Additional file 4, the canonical splice boundaries (GT/AG in introns) were implicated. These novel isoforms showed that they were processed with introns deletion. The gene structures (Figure 3) of two examples further confirmed that the RT-PCR products were derived from processed mature mRNAs, but not unspliced precursor mRNAs. On the other hand, the clone ID tracing analysis (see above) also revealed that the novel transcripts were derived from processed mature mRNAs.

Polyadenylation usually requires a hexamer motif as a primary 3'-end processing element, which is usually called the polyadenylation signal (PAS). A 50 nt nucleotide region preceding the potential cleavage sites of all 17,201 ESTs was searched for the motifs to match at least one of the thirteen known PAS hexamers (AATAAA, ATTAAA, TATAAA, AGTAAA, AAGAAA, AATATA, AATACA, CATAAA, GATAAA, AATGAA, TTTAAA, ACTAAA, AATAGA) [11]. As a result, about 65% (7,051/10,844) of all the 3'-end exons had at least one of these PAS hexamers (Additional file 1). Among 2,957 (27%, 2,957/10,844) 3'-end sites having mRNA hits (see above, Table 1 and Additional file 1), also about 63% (1,864/2,957) had at least one of thirteen above mentioned PAS. It suggests from above analysis that the novel transcript variants be derived from processed mature mRNAs, but not unspliced precursor mRNAs or degradation products of pre-mRNA.

### Novel transcript variants are truncated and missing functional domains

Intronic poly(A) sites often lead to truncated isoforms that lose important functional domain or localization signals. To evaluate if domains are lost in the novel transcript variants from intronic poly(A) sites, all protein products containing the intronic poly (A) sites had been annotated. Domains were deleted or truncated in transcript variants from 7,641 poly(A) sites from 4,142 genes (Tables 1 and Additional file 5). The detailed information of involved domains in Additional file 5 was shown in Additional file 6. Among all poly(A) sites, 1,616 could lead to deletion of trans-membrane domain. As an example, the novel isoform for TNFRSF1A (Tumor necrosis factor receptor superfamily, member 1A, also known as TNF-R1 or p55 TNFR), herein designated as TNFRSF1Aβ as it represents the second isoform of TNFRSF1A, was analyzed.

**Figure 4:**
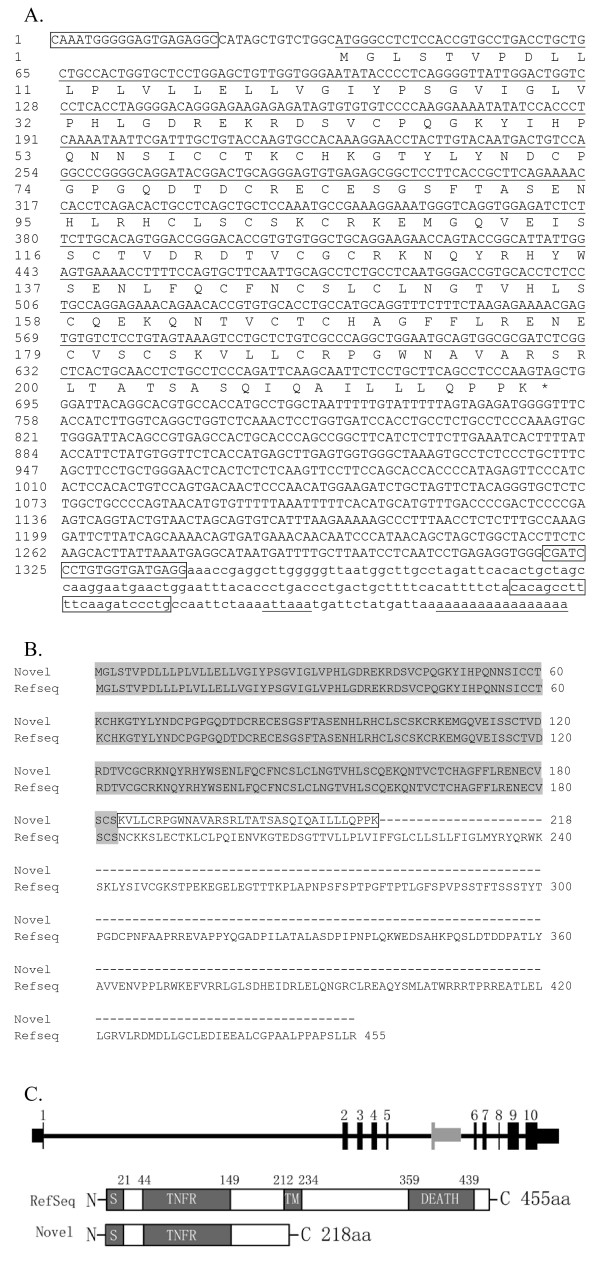
**Nucleotide and deduced amino acid sequences and the genomic structure of human TNFRSF1A beta**. (A) Nucleotide and deduced amino acid sequences of TNFRSF1A beta. The PCR amplified sequences (upper case) and 3' prolonged untranslated region (lower case) are indicated. The 657 uppercase nucleotides (underlined) are the open reading frame of TNFRSF1A beta. The boxed uppercase letters represent the second set primers, while the boxed lowercase is the first set downstream primer. The underlined lowercase letters, "attaaa", are the putative polyadenylation signal. The lowercase poly(A) tail is also underlined. The nucleotide sequence of TNFRSF1A beta has been submitted to GenBank with the accession number EU927389 [GenBank:EU927389]. (B) Amino acid sequence alignment of the novel isoform of TNFRSF1A (TNFRSF1A beta) with TNFRSF1A RefSeq [GenBank:NP_001056]. The boxed region indicates the amino acid residues specific for TNFRSF1A beta. (C) The genomic structure and organization of functional domains of TNFRSF1A beta. Exons are numbered 1 to 10 at the top. Coding exons are represented by blocks connected by horizontal lines representing introns. The 5' and 3' untranslated regions (UTRs) are displayed as thinner blocks on the leading and trailing ends of the aligning regions. The 3'-end exon (grey block) for of TNFRSF1A beta is located between exon 5 and 6. The signal peptide (S), tumor necrosis factor receptor domain (TNFR), and transmembrane helix (TM) and death domain (DEATH) are indicated. Domain regions are numbered.

TNFRSF1A is a death receptor with two known ligands, tumor necrosis factor and lymphotoxin-α. Through interactions with these ligands, TNFRSF1A initiates cellular signals and regulates many cellular functions including inflammation, immune response, proliferation, and apoptosis [47-50]. The length of PCR product is 1,339 bp which contains an open reading frame of 657 bp (Figure 4A). TNFRSF1Aβ consists of 218 amino acids (Figure 4A and 4B), and is generated from an intronic "hidden exon" between exon 5 and exon 6 (Figure 4C).

TNFRSF1Aβ lacks the trans-membrane helix and the full cytoplasmic region including the DEATH domain compared to the full-length protein (Figure 4C), while retaining the signal peptide and the conserved binding domain, that is TNFR (TNF receptor) domain.

Soluble TNFRSF1A, which functions as natural inhibitors for tumor necrosis factor, was observed and widely investigated [51-55]. Soluble TNFRSF1A is likely produced when TACE (tumor necrosis factor-alpha converting enzyme), a metalloprotease that cleaves transmembrane proteins, cleaves the TNFRSF1A ecodomain [56-58]. However, TNFRSF1Aβ we found is a natural transcript and likely encodes a secretory protein product, and may play a regulatory role preferentially by competitively binding TNFR ligand (TNF).

As alternate poly(A) sites may be regulated in a tissue- or disease-specific pattern [59,60], in addition to domain annotation, expression profiles for novel 3'-end exon sites were provided (Additional file 7). We compared the EST distribution in normal and cancerous tissues for each cluster, it revealed that some transcript variants may be cancer-specifically expressed. Moreover, an additional supplemental file (Additional file 8) provided all the candidate poly(A) sites of each human genes, integrating PolyA_DB2, Muro *et al*'s [46] and our results. Totally 112,074 sites of 19,748 genes were included.

## Discussion

In this genome-wide analysis, we showed that alternative polyadenylation in intronic sites can generate lots of novel transcript variants. We preferentially selected intronic single-block ESTs for analysis in that these ESTs were not well considered in previous studies [33-37], including Lee's research [32]. So, our work is a good complement for previous study [17,32]. Single-block ESTs within the intergenic region were not included in our analysis though some of them represent gene extensions [61]. Single-block ESTs are often suspected as contamination of genomic DNA. However, in our analysis, we showed that about 84% of the EST clusters were supported by at least one evidence: hit from full-length cDNA, multiple-block 5'-end ESTs, overlapping with transcribing sites from Affymetrix tiling array, or having multiple supporting ESTs. So by carefully screening, the single-block ESTs could be used as valuable resources for discovering novel transcripts. Besides focusing on single-block ESTs, the pipeline in our analysis was designed to improve poly(A) site detection, all these contribute to the discovery of novel intronic 3'-end exons. During our analysis, we found that more than 90% of the EST entries in our results were created before the polyA_DB2 was released. It implied that most of the novel transcript variants were derived by the improvement of our detection methods and the consideration of single-block ESTs, but not merely by the growth of the transcript databases.

Although different methods have been used for poly(A) site prediction [10,62], current methods achieve only moderate sensitivity and specificity. For example, about 47% of known poly(A) sequences in the polyA_DB database were not predicted the Support Vector Machine (polya_svm) [10]. Among our predicted 3'-end exon sites, less than thirty can be predicted by polya_svm (threshold = 0.5 when the genomic region containing the poly(A) cluster region ± 300 nucleotides was used for predictions). However, 68% of the 17,201 ESTs, which correspond to about 63% of the 10,844 3'-end exons (Additional file 1), have at least one of thirteen known PAS hexamers. This low detection rate of prediction by polya_svm likely results from heterogeneity of the intronic poly(A) sites compared to the conventional 3'-most poly(A) sites.

It is worthy of note, a method different to ours for identification of 3'-ends of genes was made according to EST frequency histogram along the genome by Muro *et al *[46]. They show that 22-52% of sequences in commonly used human and murine "full-length" transcript databases may not currently end at bona fide polyadenylation sites. Since the average length of the 3'-end exons of all the current human RefSeqs is about 820 nucleotides, they will get longer according to Muro *et al *'s results. As the comparison in the text has shown, Muro *et al*'s method and ours have respective advantages, and complement each other. Both methods will contribute to identification of full-length transcripts.

Novel 3'-end exons we detected could be defined as "hidden exons" and "composite exons" described previously [19]. However, some apparent "hidden exons" could be actually "composite", because ESTs only represent partial cDNA sequences and may be extended to overlap with known exons.

Not all intronic poly(A) sites correspond to actual novel transcript variants. For example, internal priming, due to a consecutive string of 'A's in the mRNAs, results in false positives. For cDNA library construction, oligo-dT is often used as the primer for first strand cDNA synthesis. This primer can anneal to the internal priming site, producing truncated sequences. Internal priming accounts for about 12% for the total 3' ESTs in the database [63]. In previous study like Tian's [11], the genomic DNA sequence around the predicted poly(A) site was checked. If there were more than 6 consecutive 'A's or at least 7 'A's in 10 nt window, it was suspected to be an internal priming site. However, when applied the criterion to study the adjacent DNA sequence of 3'-end of human RefSeq mRNAs, it was found that 19.4% (6,147/31,642) mRNAs had such A trait at their 3'-ends. So if using the above criterion, many true positive sites might be missed. In our analysis, we tried to reduce internal priming sites by eliminating all ESTs that could be aligned well with known RefSeq mRNAs (see Methods).

In order to find novel transcript variants as many as possible, we did not request an accurate signature of exon junction and cleavage site. This is different to those previous reported [17,19,30-32]. The 3'-end exon site provides the approximate locus of the "composite exons" or the "hidden exons" for novel isoforms. The supporting ESTs of a 3'-end exon site further provide proper sites for downstream primer designing to amplify the full coding region of corresponding novel isoforms. We performed RT-PCR to validate some interested candidates with success rate of about 38% (10/26, see Results). Sequence analysis revealed they were derived from processed mature mRNA, but not unspliced precursor.

In our analysis, although most of the sites are supported by at least two types of evidences, there are still 1,468 sites containing only one EST sequence without supporting in other way. Some of these sites may truly represent novel transcript variants associated with low expression levels. For example, the sites DB550185 (ExonSiteNo: 8501), DB347581 (ExonSiteNo: 8549), DB536313 (ExonSiteNo: 8628), and DB517750 (ExonSiteNo: 9840), and DB512524 (ExonSiteNo: 10422), they contain only one EST sequence, but the EST is from a full-length cDNA clone (Additional file 1 and 3).

One type of RNA polyadenylation controls RNA degradation in the nucleus [64-66]. The exosome plays a key role in the surveillance of nuclear mRNA synthesis and maturation. Poly(A) tails guiding RNA to be degraded by the exosome are usually shorter than those increasing mRNA stability, and these poly(A) tails are not made strictly of 'A's. These sites were not actively eliminated in our analysis, but they are unlikely to greatly affect the results because they would not be detected under our stringent criteria. On the other hand, sequence analysis of the poly(A/T)-tailed ESTs revealed that PAS did exist in most of our ESTs. This result combined with other evidences, suggest our predicted poly(A) sites should represent bona fide mRNAs, but not unspliced precursor mRNAs, neither the degradation products.

Another type of RNA quality control is nonsense-mediated mRNA decay (NMD), which selectively degrades mRNAs that contain a premature translation termination codon (PTC, also called "nonsense codon") [67,68]. Although NMD mainly acts as quality control to eliminate faulty transcripts in gene expression, it is also involved in physiological and pathological functions [68,69]. Usually, NMD occurs when translation terminates more than 50-55 nucleotides upstream of the exon-exon junction, in which case components of the termination complex are thought to interact with the exon-junction complex (EJC) to elicit NMD [67]. Although 45% of alternatively spliced mRNAs are predicted to be an NMD target [68], an mRNA is immune to NMD if translation terminates less than 50-55 nucleotides upstream of the 3'-most exon-exon junction or downstream of the junction. This means if a natural stop codon of an mRNA exists in the 3'-end exon, it is not subject to NMD. The transcripts predicted in our study use an alternative 3'-UTRs, assuming that upstream exons do not change. Because we have not got the full-length form for each transcripts, we can not estimate the proportion of our results that would be affected by NMD. However, it has been reported that alternative polyadenylation may be an NMD-rescue regulatory mechanism in PTC-containing mRNAs [70]. Our data seem to be consistent with the view. Actually all the novel transcripts proved by RT-PCR experiments in our study comprise the natural stop codon in the last exon. A further analysis revealed that in nearly all the 3'-end ESTs except some very short ones, stop codons exist in all three reading frames (data not shown). So if there were no correct stop codons in the 5'-exons, the stop codon in the 3'-end exons of our result would be used. This is different to middle exons that may not contain in-frame stop codons and could not help conveniently clone transcripts with complete coding regions.

It should be noted that a large number of non-coding RNAs (ncRNAs) are expressed from the mammalian genome [71,72]. These ncRNAs include miRNAs, snoRNAs, snRNAs, and piRNAs, and so on, which are involved in controlling various levels of gene expression in physiology and development. Non-coding RNAs can be derived from antisense or sense transcripts with overlapping or interlacing exons, or retained introns. To investigate that whether the internal intronic transcripts in our data actually represent known ncRNAs, we compared the chromosome alignment position between the 3'-end exon sites in our study and those of human ncRNAs from NONCODE v2.0 [72]. In 35,2434 human ncRNA entries collected in NONCODE v2.0, less than one hundred 3'-end exon sites were overlapped (data not shown). So it seems that most of our 3'-end exons do not represent known ncRNAs. Whereas, we found many poly(A) sites were located in the introns before the coding exons. If they were real, the potential novel transcripts would be composed of the 5'-UTR of the original mRNA. Whether the transcripts encode small ORFs or regulatory small RNAs needs to study in the future.

## Conclusion

In conclusion, our results identify novel 3'-end alternative splicing isoforms. The expression of these novel variants was confirmed with computational and experimental tools. These data provide a genome-wide resource for identification of novel human transcript variants with intronic polyadenylation sites, and offer a new view into the mystery of the human transcriptome.

## Methods

### Data source

The University of California, Santa Cruz (UCSC) Genome Browser Database (GBD) http://genome.ucsc.edu provides a common repository for genomic annotation data, including comparative genomics, genes and gene predictions, mRNA and EST alignments, and so on [73,74]. The human EST annotation file (all est), RefSeq mRNAs, all known mRNA sequences, and RefSeq annotation files were downloaded from the UCSC bioinformatics web site (April 2008 version). ESTs were downloaded from the NCBI dbEST database (ftp://ftp.ncbi.nih.gov/repository/dbEST/, April 2008 version). The UniGene database was downloaded from ftp://ftp.ncbi.nlm.nih.gov/repository/UniGene/. Sequences were aligned by using BLAT program http://genome.ucsc.edu/cgi-bin/hgBlat. Perl scripts were used for data extraction and analysis. The BED files in NONCODE v2.0 about human non-coding RNAs were downloaded from http://www.noncode.org/download.htm. The annotated chromosomal positions were used for overlapping analysis with 3'-end exon sites.

### Intronic 3'-end exon site identification and EST clustering

To identify novel transcript variants, we focused on intronic 3'-end exon sites. The outline of data analysis is shown in Figure 1.

First, single-block ESTs were collected from UCSC Genome Browser annotation file. The annotation file provides detailed information including chromosome localization, transcription direction, blockCount (number of blocks in the alignment) and blockSizes (comma-separated list of sizes of each block). BlockCount loosely reflects the alignment exon number. BlockCount increases as EST quality decreases. Many ESTs were annotated for multiple blockcounts but are really single-block ESTs. To identify all single-block ESTs, we corrected for misplaced blocks as follows: if the chromosomal distance between consecutive blocks was less than 10 nucleotides or if the chromosomal distance was more than 10 nucleotides but the blocksize was less than or equal to 10 nucleotides, the blockcount was reduced by one. If the final blockcount was one, the EST was kept as a single-block EST.

Second, 3'-end exon sites were identified by a poly(A/T)-tail. All single-block ESTs were checked for 5'-end 'T's or 3'-end 'A's as poly(A) tails in the reverse and forward orientations, respectively. The EST was firstly requested to contain at least 10 consecutive 'A's or 'T's in either terminal 100 nucleotides. Then poly(A/T) tail was determined if one of the following criteria was satisfied: (1) if($seq = ~/^t{0,} [atgcn]{0,5}t{10,}/i || $seq = ~/a{10,} [atgcn]{0,5}a{0,}$/i || $seq = ~/^t{0,} [atgcn]{0,10}t{12,}/i || $seq = ~/a{12,} [atgcn]{0,10}a{0,}$/i || $seq = ~/^[atgcn]{0,2}t{2,} [atgcn]{0,2}t{8,}/i || $seq = ~/a{8,} [atgcn]{0,2}a{2,} [atgcn]{0,2}$/i){...}; (2) the EST had 20 or more consecutive 'A's or 'T's in either terminal 100 nucleotides, or the EST had 40 or more consecutive 'A's or 'T's in the entire sequence; (3) the EST had more than 15 'A's or 'T's within a 20 nucleotide window in either terminal 50 nucleotides. The criteria (1) was the most effective and could identify most of poly(A) tails. More consecutive 'A's or 'T's were needed if interrupted by other nucleotides because of sequence quality. On the other hand, various length of vector sequences are contained in some proportion of ESTs, and the poor sequencing quality in the ends or linker sequences in oligo(T) primers should be concerned, therefore, criteria (2) and (3) were introduced. To our knowledge, the distance from sequencing primers to multiple cloning site (MCS) is not too long and 100 nucleotides were used as threshold. These criteria could provide suitable endurance for sequence quality. The chromosomal loci for these poly(A/T)-tailed ESTs locations were regarded as 3'-end exon sites. The remaining ESTs were considered as non-poly(A/T)-tailed ESTs. Non-poly(A/T)-tailed ESTs were used as supporting evidence for novel transcript variant expression if their chromosome alignment overlapped with poly(A/T)-tailed ESTs.

Third, the poly(A/T)-tailed EST candidates were used as queries to blast the RefSeq mRNA database. The E-value was set at e^-10^. All ESTs with a hit were removed. The remaining ESTs were further blasted against all mRNA database with the same E-value to provide transcriptional evidence.

Finally, the ESTs were mapped to genes. The transcriptional orientation of a gene was annotated in the downloaded file "refSeqAli.txt.gz". The orientation of the EST sequences relative to their mRNA was determined by the presence of a 5'poly(T) tail or a 3'poly(A) tail. If both poly(A) and poly(T) tails existed in the same EST, overlapping poly(A/T)-tailed ESTs were used to determine the true orientation. Poly(A/T)-tailed ESTs and non-poly(A/T)-tailed ESTs were clustered according to their chromosomal alignments. The start and end positions for each cluster were recorded as the position of the 3'-end exon site. The RefSeq gene corresponding to each cluster was determined. Although many genes have more than one RefSeq, we always selected the same RefSeq for clusters from the gene, unless the EST alignment was not within that RefSeq locus. All the ESTs were analyzed for their tissue source and divided into cancer-originated or normal-originated.

### Tracing sequences via clone IDs

All clone IDs were extracted by EST accession numbers. For each clone ID, the opposite end sequence was traced. For each end sequence, the GT/AG splicing boundary determined the transcriptional orientation. If the traced sequence had both the same transcription orientation as the RefSeq mRNA and at least one overlapping alignment block, the EST clone represented a novel isoform.

### Comparison with Affymetrix genomic tiling array data

The transcription fragment file of Affymetrix genomic tiling array were downloaded from the UCSC Genome Browser http://hgdownload.cse.ucsc.edu/goldenPath/hg18/database/. The chromosomal location of the fragments was compared with the 3'-end exon sites. If the fragments overlapped a 3'-end exon site, the EST represented a novel transcript variant.

### RT-PCR experiments

RT-PCR experiments were made to clone some interested transcript variants. Nested-PCR was performed. The primers were shown in Additional file 4. The cDNA template was the Clontech mixed human multiple tissue cDNA panel, including ten human tissues (brain, spleen, heart, skeletal muscle, thymus, liver, pancreas, lung and placenta and kidney). The Touchdown-PCR method had the following conditions: denaturing for 30 s at 94°C; annealing for 30 s from 65°C to 60°C, decreasing at 0.5°C each cycle, for the first 10 cycles and at 60°C for the last 20 cycles; extension for 90 s at 72°C for all cycles, with the final extension at 72°C for 5 min. Each experiment was done in a 20 μl PCR reaction volume, containing 2 μl of template, with a GeneAmp^® ^PCR System 2700 amplifier. Conditions for the second PCR were the same, except that 3 μl of template derived from the first PCR products were used. The second PCR products were for electrophoresis and recovered, then cloned in pGEM-T easy vector (Promega) or directly sequenced. The sequences were aligned with the BLAT, ClustalW http://www.ebi.ac.uk/clustalw/, and BLAST.

### Comparison with other alternative splicing related databases

The sequences of human alternative splicing variants were downloaded from the ASAP II http://bioinfo.mbi.ucla.edu/ASAP2/ and ASTD http://www.ebi.ac.uk/astd databases. The poly(A/T)-tailed EST candidates were used as queries to search these databases by blast program. The E-value was set at e^-10 ^and minimum match of 60 nt with 80% identity was requested. A comparison between our and Muro *et al*'s 3'-terminal sequence data was also made using blast analysis. The accession numbers of poly(A/T)-tailed ESTs as well as the positions of chromosome alignment of each cluster were used for comparison with the PolyA_DB2 database. The ESTs without hits represent novel 3'-end exons.

To supply a comprehensive list of poly(A) sites, we integrated the PolyA_DB2, Muro *et al*'s [46] and our prediction (Additional file 8). The integration was done according to the chromosomal location of predicted sites. Sites that were within 20 nt to each other were taken as one cluster. For each site, the strand that the site belongs to was determined by the direction of known mRNA containing the site. Sites those were aligned to random chromosome were eliminated.

### Domain mapping

Most intronic poly(A) sites result in changes in CDS region [17]. To determine the effects of these CDS changes, we mapped domains in all the potential novel transcript isoforms with the assumption that the exons before the novel poly(A) site remain unchanged. Domain information was extracted from RefSeq. The secretory signal and trans-membrane helix were analyzed with SignalP http://www.cbs.dtu.dk/services/SignalP/ and TMHMM http://www.cbs.dtu.dk/services/TMHMM/, respectively.

### Internal priming site evaluation

We downloaded the alignment data of human RefSeq mRNA from UCSC Genome Browser. The -10 to +10 genomic DNA sequence around the 3'-end was extracted. If there were more than 6 consecutive 'A's or at least 7 'A's in 10 nt window, it was taken as a 'A' trait.

In our methods for poly(A) identification as mentioned in above criteria, especially, the criteria (3), it was likely to introduce internal priming sites. To try the best to decrease the false positive results, validation tests (see above) were performed for the 3'-end exon candidates by blast analysis against all known mRNA database, tracing EST clone ID, RT-PCR experiments and comparison with Affymetrix genomic tiling array data. The 3'-end exon sites validated by the first three processes produced more reliable results than those validated only by Affymetrix transcriptional fragments or not because of exon overlapping with the containing genes. Therefore, if the 3'-end exon candidates were not supported by any of the first three validation tests, all the poly(A/T)-tailed ESTs in these 3'-end exon candidates were re-analyzed, and an extra criterion was introduced, i.e. the sequence downstream the poly(A) sites should not match the corresponding genomic region as to eliminate internal priming sites as possible as we can. For this purpose, we compared two types of positions, the EST alignment 3'-end position in chromosome and the identified poly(A) site. If their distance was within 20 nt, the corresponding poly(A/T)-tailed EST was kept, otherwise it was abandoned. Moreover, if all the poly(A/T)-tailed ESTs were completely matched the genome, or at most with 5 nt hanging tails without matching, the containing 3'-end exon sites were deleted.

## Authors' contributions

PW and PY participated in the design of the study, carried out the bioinformatic analysis. PW and PG performed the validation assays. PW wrote the manuscript. PY, TS and DM were involved in the conceptualization and writing. All authors read and approved the final manuscript.

## Supplementary Material

Additional file 1 **Summary information for the 10,844 3'-end exon sites**. In sheet "all_site" lists the summary information for the 10,844 3'-end exon sites, whereas in sheet "validation_test" lists more reliable sites from the validation tests, blast analysis against all known mRNA database, EST clone ID analysis, and RT-PCR experiments. The headings in sheet "all_site" are defined as follows: "ExonSiteNo" is the serial number for the 3'-end exons; "AccNo" is, in most cases, the first sequence in each poly(A) cluster; "Chr" is the aligned chromosome for each site; "StartPos" is the start position of the chromosome alignment; "EndPos" is the end position for each cluster; "RefSeq" is reference sequence; "Definition" is the UniGene name for each RefSeq; "Symbol" is the UniGene symbol. "Locuslink" is the gene-based ID number; "SiteEST" is the list of poly-(A/T)-tailed ESTs in each cluster; "SeqCount" is the number of sequence counts in SiteEST; "If_BlastHit" marks whether there is a blast hit in the all known mRNA database; and "If_PAS" marks whether there are polyadenylation signals in poly(A/T)-tailed ESTs. "If_PolyA_DB2" marks whether this is overlapping sequence in PolyA_DB2. "If_PolyA_DB2" is true if the 3'-end ESTs are in PolyA_DB2 database or the 3'-end exon site overlaps a site in PolyA_DB2 database. "If_ASAPII", "If_ASTD" and "If Muro *et al*'s data" mark whether this is overlapping sequence in ASAP II, ASTD and Muro *et al*'s databases, respectively. "If_50aa" marks whether the 3'-end exon is behind the position of first 50 amino acid residues encoded by the RefSeq. The same headings in sheet "validation_test" have the same meaning as in sheet "all_site". "RT-PCR" marks whether the 3'-end exon is validated by RT-PCR experiments. "CloneIDSource" marks whether the EST clones are derived from poly(A/T)-tailed ESTs or non-poly(A/T)-tailed ESTs. "ExonSiteNo", "Locuslink", "Symbol", and "Definition" have the same meaning in subsequent tables. Click here for file

Additional file 2 **Supporting evidence for all 3'-end exon sites**. The headings are defined as follows: "SupportESTs" are the supporting ESTs for each sites; "SupportCount" is the number of sequence counts in "SupportESTs"; "AffyArraySupport" reveals which cell lines support each site and their expression fragment number; "AffyCount" is the total number of detected expression fragments in all cell lines; and, "CloneIDSupport" are the corresponding clone IDs to the "SupportEST" that are used for tracing end sequences. If the traced sequences have multiple blocks that align with the genome and these blocks overlapped with known reference sequence for the same genes, "CloneIDSupport" is recorded. For each site, the serial position is separated into three parts, the clone and both end sequences, respectively. "CloneCount" is the total number of clones in "CloneIDSupport". "If_BlastHit" marks whether there is a blast hit in the all known mRNA database. Click here for file

Additional file 3 **Novel transcript variants represented by clone ID**. Novel transcript variants and their respective EST clones are listed. The headings are defined as follows: "Clone ID" is the clone ID for the novel transcript; "AccNo(5'-end)" and "AccNo(3'-end)" are the accession numbers of the 5'-end and 3'-end sequences from the same clones, respectively; "SiteType" is the pattern of alternative polyadenylation relative to the reference sequence; "PolyA_DB2" identifies if the 3'-end ESTs are in PolyA_DB2 database; and "AssSeq" are the assembled sequences for each clone. The upper and lowercase indicate the 5'-end and 3'-end sequences, respectively. "AssSeq" does not represent the full sequences for the same EST clones. "SiteType", "AccNo" and "PolyA_DB2" have the same meaning in subsequent tables. Click here for file

Additional file 4 **Amplification of novel transcript variants**. The headings are defined as follows: "AmpSeq" is the amplified cDNA sequence; "PCRlength" is the PCR product length; "ProteinSeq" is the putative protein product from the "AmpSeq"; "ProLength" is the protein length in "ProteinSeq"; and "FirstPrimer(5')", "FirstPrimer(3')", "SecondPrimer(5')" and "SecondPrimer(3')" are the first and second primer sets, respectively. All the primer sequences are given in 5' to 3' orientation. Click here for file

Additional file 5 **Domain analysis involved in all sites**. The headings are defined as follows: "If_Secretory" is true if SignalP predicts a signal peptide; "TM_List" is the list of trans-membrane helices predicted in TMHMM; "TM_Intact" are transmembrane helices retained in the novel transcript variants; "TM_deletion" are deleted transmembrane helices in the novel transcript variant; "ForeDomain" and "PostDomain" indicate whether the deleted domains are just before or after the 3'-end site, and "InterruptedDomain" indicates that the domain is divided by the site; "If_DomainDeletion" is true if at least one domain is deleted in the novel transcript variant. Click here for file

Additional file 6 **Domain annotation**. The headings are defined as follows: "PSS_ID" is a unique ID from position-specific scoring matrices; and "CD_accession", "CD_name", and "Description" are the accession number, the name of conservative domain, and the domain description, respectively. Click here for file

Additional file 7 **Tissue distribution of poly(A/T)- and non-poly(A/T)-tailed ESTs**. The headings are defined as follows: "Total_tissue" describes all tissue (including cancer) that had EST expression and their corresponding EST counts; "Total_tissue_acc" are the accession numbers for tissue types in "Total_tissue"; accession numbers for the same tissue are separated by a comma; "Cancer_tissue" and "Cancer_tissue_acc" are the same as "Total_tissue" and "Total_tissue_acc" with ESTs derived from cancer tissues; "Cancer/total" shows the ratio of cancerous ESTs to all ESTs; "Cancer_vs_normal" is the difference when the cancerous EST count is subtracted from normal tissue EST count; "AffyArraySupport" from Additional file 2 is shown as evidence for expression. Click here for file

Additional file 8 **An integrated poly(A) sites of human genes**. The headings are defined as follows: The 1-5th columns are the chromosome, site position, strand, gene symbol and Entrez Gene ID. The 6-8th columns are the poly(A) site number or representative supporting ESTs from PolyA_DB2, Muro *et al*. and our prediction, respectively. The 9th column stands for if there is PAS in the upstream of the site. Some sites from PolyA_DB2 have no corresponding mRNA sequences thus can not determine the "strand" information. Click here for file
